# Type of Referral, Dialysis Start and Choice of Renal Replacement Therapy Modality in an International Integrated Care Setting

**DOI:** 10.1371/journal.pone.0155987

**Published:** 2016-05-26

**Authors:** Belén Marrón, Janusz Ostrowski, Marietta Török, Delia Timofte, Attila Orosz, Andrzej Kosicki, Alicja Całka, Daniela Moro, Dezider Kosa, Jenö Redl, Abdul Rashid Qureshi, Jose Carolino Divino-Filho, d.PD Clinics Eastern Europe

**Affiliations:** 1 Diaverum Home Therapies, Medical Office, Munich, Germany; 2 Wloclawek Diaverum Clinic, Wloclawek, Poland; 3 Szeged Diaverum Clinic, Szeged, Hungary; 4 Semaparc Diaverum Clinic, Bucharest, Romania; 5 Bajcsy Diaverum Clinic, Budapest, Hungary; 6 Przemysl Diaverum Clinic, Przemysl, Poland; 7 Olsztyn Diaverum Clinic, Olsztyn, Poland; 8 Sibiu Distributei Diaverum Clinic, Sibiu, Romania; 9 Zalaegerszeg Diaverum Clinic, Zalaegerszeg, Hungary; 10 Szolnok Diaverum Clinic, Szolnok, Hungary; 11 Division of Renal Medicine, CLINTEC, Karolinska Institute, Stockholm, Sweden; Hospital Universitario de La Princesa, SPAIN

## Abstract

**Introduction:**

Integrated Care Settings (ICS) provide a holistic approach to the transition from chronic kidney disease into renal replacement therapy (RRT), offering at least both types of dialysis.

**Objectives:**

To analyze which factors determine type of referral, modality provision and dialysis start on final RRT in ICS clinics.

**Methods:**

Retrospective analysis of 626 patients starting dialysis in 25 ICS clinics in Poland, Hungary and Romania during 2012. Scheduled initiation of dialysis with a permanent access was considered as planned RRT start.

**Results:**

Modality information (80% of patients) and renal education (87%) were more frequent (p<0.001) in Planned (P) than in Non-Planned (NP) start. Median time from information to dialysis start was 2 months. 89% of patients started on hemodialysis, 49% were referred late to ICS (<3 months from referral to RRT) and 58% were NP start. Late referral, non-vascular renal etiology, worse clinical status, shorter time from information to RRT and less peritoneal dialysis (PD) were associated with NP start (p<0.05). In multivariate logistic regression analysis, P start (p≤0.05) was associated with early referral, eGFR >8.2 ml/min, >2 months between information and RRT initiation and with vascular etiology after adjustment for age and gender. “Optimal care,” defined as ICS follow-up >12 months plus modality information and P start, occurred in 23%.

**Conclusions:**

Despite the high rate of late referrals, information and education were widely provided. However, NP start was high and related to late referral and may explain the low frequency of PD.

## Introduction

The prevalence of chronic kidney disease (CKD) defined as eGFR <60 ml/min/1.73 m^2^ has reached epidemic proportions, with studies showing a prevalence of 10–13% [1–3]. Indeed, CKD is recognized as a growing global public health problem due to the rising rates of diabetes mellitus, obesity, hypertension and aging populations [4–6]. The cost associated with renal replacement therapy (RRT) [dialysis or kidney transplantation] needed by these patients (roughly 0.1% of the general population), comprises 1–2.5% of the total health care spending in high-income countries [7]. The variation in RRT incidence across countries is thought to be associated with countries’ economics, health care system and renal service factors rather than population demographics and health status [7–8].

Some traditional hemodialysis (HD) providers have recently developed ICS clinics aiming to increase quality of life and life span for patients as well as to diminish costs through a more sustainable renal care model [9–10]. ICS offers a holistic renal care approach to patients in the transition from early CKD care into RRT, offering at least both types of dialysis (HD and PD). These ICS clinics usually offer a multidisciplinary team approach, including dietitians, psychologists and social workers, and providing information, education and support to revitalize these patients in all functional areas [11]. ICS may increase efficiency of CKD care by promoting timely and adequate channels for patient referral to nephrologists, contributing to a planned dialysis start and offering balanced high quality RRT modality information as well as education [11–12]. In order to diminish the gap between reality and the desirable care needed, several pitfalls should be addressed: inadequate medical training, timely referral to nephrologists, inappropriate patient information and education for RRT modality choice, lack of specialized predialysis programs and lack of planned RRT initiation [13].

In addition, PD remains underused despite having demonstrated to be at least equal to HD as the first dialysis modality, especially while there is residual renal function [13–17]. Specialized predialysis programs have consistently demonstrated important benefits such as delayed progression of renal insufficiency, improved patient outcomes, decreased hospitalizations and urgent dialysis initiation need, as well as increased patient participation in modality choice and thereby increased use of home therapies [18–25]. However, such infrastructures are not widely established and frequently insufficiently staffed [13,19,23,26–29].

In the present study, we assess in a group of Eastern Europe ICS clinics which factors determine type of referral, modality provision and dialysis start on final RRT of a private renal services provider (Diaverum).

## Materials and Methods

This is an international-multicenter observational retrospective study on the impact of ICS in all consecutive patients who started maintenance dialysis for CKD-5 from 1^st^ January through 31^st^ December 2012 in twenty-five ICS clinics in Poland, Hungary and Romania. Patients with pre-emptive transplants were excluded from the study.

Information was collected on demographic variables, cause of renal disease, follow up since diagnosis of kidney disease, medical specialist providing care, type of referral to ICS clinic [defined as early (ER) if ≥ 3 months and late (LR) if <3 months], predialysis care devoted by general nephrologist or by specialized predialysis staff (where at least a nephrologist and a nurse have been appointed part time into specific predialysis care), number of medical visits in the year prior to the start of dialysis, type of dialysis at first session and as ascribed chronic RRT, analytical parameters at dialysis start [24 h. urine creatinine clearance, estimated GFR (MDRD-4), serum creatinine, albumin, calcium and phosphorus, hemoglobin levels] and EPO prescription.

Information to patients on RRT modality (if provided) and general renal education (if delivered) were analyzed in a qualitative manner. Patients were assigned to the "modality informed" group when different RRT modalities were explained by staff, supportive information tools were used for this purpose (e.g. brochures, DVDs) or meetings with other patients in clinic facilities took place. Renal education was considered to be provided when patients were taught how to care for renal disorders and about the importance of compliance with prescriptions and follow-up visits. No single common protocol was created for this purpose. Each clinic designed the type and content of information taking into account local cultural issues.

The patient choice of dialysis modality, informed consent signing (for information and at dialysis start) and time elapsed from provision of information to dialysis start were also recorded.

RRT start was considered non-planned (NP) when either functional permanent access was lacking or an unscheduled (urgent) start occurred, even if a permanent dialysis access was in place. Optimal care was defined as patients followed-up in an ICS with more than 12 months receiving RRT modality information and having a planned dialysis start.

### Ethics

This is a retrospective, non-interventional, observational cohort study with sourcing data obtained from routine practice in Diaverum clinics located in Romania, Hungary and Poland during 2012. The Study was approved by the Quality, Compliance and Data Protection Institution’s Commissioner. Patient records were anonymized and de-identified prior to analysis. Participant patients signed an informed consent form that included providing permission to record data for research and publication purposes in an anonymized manner.

### Statistical Analysis

Data are expressed as median (10th to 90th percentile) or percentage, as appropriate. Statistical significance was set at the level of p <0.05. Comparisons between two groups were assessed with the nonparametric Wilcoxon test for continuous variables and a chi-square test for nominal variables. Differences among three or more groups were analyzed using the nonparametric ANOVA Kruskal–Wallis test. Spearman rank correlation analysis was used to determine associations between continuous and ordinal variables. Multivariate logistic regression analyses were used to assess determinants of P and ER vs. NP start, data was expressed as Odd ratios and 95% CI. The covariates were selected on the basis of biological plausibility. All statistical analyses were performed using statistical software SAS version 9.4 (SAS Campus Drive, Cary, NC, USA).

## Results

**Fig 1:**
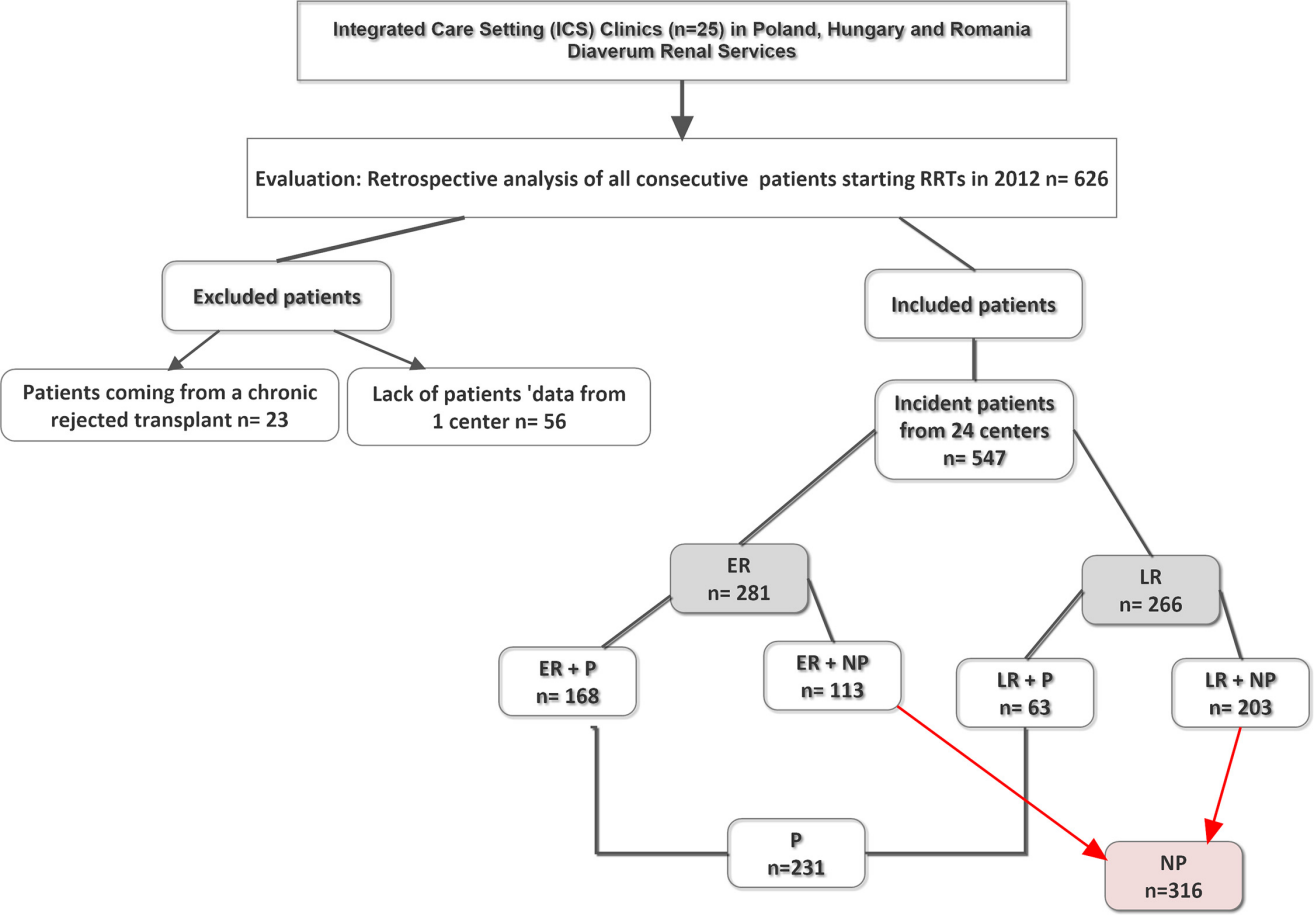
Patients Flowchart for clinical study evaluation. Abbreviations: ER, early referred patients; LR, late referred patients; P, planned dialysis start patients; NP, non-planned dialysis start patients; ER+P, early referral and planned patients; ER+NP, early referral and non-planned patients; LR+P, late referral and planned patients; LR+NP, late referral and non-planned patients. A total of 626 patients started dialysis in 2012 from 25 Integrated Care Setting Clinics in Poland, Hungary and Romania at Diaverum Renal Services but only 547 were evaluated after excluding patients returning from a previous kidney transplantation (n = 23) and from one center with incomplete data (n = 56). Evaluated patients were primarily divided into two groups according with type of referral being 281 patients ascribed to the early referral and 266 patients into the late referral. Both groups were secondarily divided into another two groups each, depending on type of dialysis start. 168 patients were considered as early referred and with a planned dialysis start, 113 patients were considered as early referred but with a non-planned dialysis start, 63 patients were considered late referred but with planned dialysis start and 203 patients were late referred and had non-planned dialysis start. Planned dialysis patients were 231 of the total population and non-planned dialysis start were 316.

A total of 626 patients started dialysis in 2012 but only 547 were evaluated after excluding patients returning from kidney transplantation (n = 23) and from one center with incomplete data (n = 56) (Fig 1). Patient classification according to type of referral and type of dialysis start was as follows: Group ER+P [168/547 (31%)]; Group ER+NP: [113/547 (21%)]; Group LR+P: [63/547 (11%)] and Group LR+NP: [203/547 (37%)]. Main clinical characteristics according to dialysis start planning are summarized in Table 1.

**Table 1:** Clinical characteristics according to planning of dialysis start.

Population	Total	P dialysis start	NP dialysis start	P-value
n	547	231	316	
Males, n (%)	332 (61)	144 (62)	188 (59)	NS
Age, years	64 (42–81)	63 (40–80)	64 (43–80)	NS
Weight at dialysis start, Kg	77 (56–100)	80 (58–97)	73 (55–101)	<0.01
**Cause of ESRD (%)**				
Diabetes mellitus, n (%)	162 (30)	74 (32)	88 (28)	<0.001
Glomerular, n (%)	64 (12)	27 (12)	37 (12)	
Inherited, n (%)	26 (4.7)	18 (8)	8 (2.5)	
Unknown, n (%)	52 (9.5)	11 (5)	41 (13)	
Others, n (%)	56 (10)	15 (6)	41 (13)	
Tubulo-interstitial, n (%)	62 (11)	19 (8)	43 (14)	
Vascular, n (%)	125 (23)	67 (29)	58 (18)	
**Biochemistry at dialysis start**				
Serum Creatinine (mg/dl)	6.1 (3.3–11.4)	5.2 (3.3–9.2)	6.8 (3.9–12.5)	<0.001
CCr 24h (ml/min)	9 (5–16)	9 (5–15)	8 (4–16)	NS
EPO prescribed, n (%)	209 (38)	115 (50)	94 (30)	<0.001
Haemoglobin (g/dl)	10 (7–12)	10 (8–11)	9 (7–11)	<0.001
Serum Calcium (mg/dl)	8.4 (7.3–9.9)	8.8 (7.4–9.9)	8.3 (7.3–9.4)	<0.001
Serum Phosphorus (mg/dl)	4.8 (3.4–7.6)	4.6 (3.4–7.4)	5.2 (3.6–7.7)	<0.001
S. Albumin (g/dl)	3.6 (2.8–4.2)	3.8 (3.2–4.3)	3.4 (2.7–4.0)	<0.001
**RRT at 1**^**st**^ **dialysis session**				
HD, n (%)	502 (91.7)	191 (82.6)	311 (98.4)	<0.001
PD, n (%)	45 (8.2)	40 (17.3)	5 (1.5)	
**1**^**st**^ **chronic RRT**				
HD, n (%)	488 (89.2)	191 (82.6)	297 (94)	<0.001
PD, n (%)	59 (10.7)	40 (17.3)	19 (6)	

Values are median (10^th^ to 90^th^ percentile), or percentage. Abbreviations: P, planned dialysis start; NP, non-planned dialysis start; ESRD, end stage renal disease; RRT, renal replacement therapy; HD, hemodialysis; PD, peritoneal dialysis; EPO, erythropoietin.

### Initial CKD care follow up, predialysis care and type of referral to ICS

The majority of the patients 459/547 (84%) were followed-up at initiation of CKD care by nephrologists (48%), general practitioners (12%) and other specialists (24%). Half (266/547) of the patients were referred late to ICS [in Romania (57%), Poland (50%) and Hungary (35%)]. Predialysis (GFR <30ml/min) care was provided in ICS more frequently by general nephrologists (68%) rather than by specialized predialysis staff (29%). Most patients 479/547 (87%) received some renal education prior to dialysis start. RRT modality information was provided to 436/547 (80%) of patients. Of the modality informed patients, final RRT was exclusively based upon patient´s choice in 57% of cases. The median time from information to dialysis start was 2 months. Patients (246/436; 57%) signed informed consents at the time of modality provision and at the time of dialysis start (421/547; 77%). More patients received modality information in the PD group (92%) compared with 78% in the HD (p = 0.02). Optimal care was observed in 123/547 (23%) of the patients.

### Planned versus non-planned start

Reasons for becoming NP and needing urgent dialysis are presented in Table 2.

**Table 2:** Reasons for non-planned start and need of urgent dialysis start.

Population	NP	ER+NP	LR+NP	P-value
n	316	113	203	
**Cause/s for urgent dialysis start**				
Asymptomatic + biochemistry abnormalities, n (%)	8 (2.5)	2 (2)	6 (3)	0.20
Over imposed acute kidney injury on CKD, n (%)	20 (6.3)	7 (7)	13 (6)	
Hyperkalemia, n (%)	5 (1.5)	3 (3)	2 (1)	
More than one cause at once (mix), n (%)	79 (25)	22 (21)	57 (28)	
Other reasons, n (%)	13 (4)	6 (6)	7 (3)	
Clinical symptoms of uremia, n (%)	126 (40)	39 (27)	87 (43)	
Volume overload, n (%)	55 (17.4)	26 (23)	29 (14)	
Unknown	10 (3)	8 (7)	2 (0.9)	
**Reasons for becoming NP**				
Acute factor deteriorating previous GFR, n (%)	27 (9)	12 (12)	15 (9)	<0.001
Mix reasons, n (%)	19 (6)	10 (10)	9 (5)	
Others, n (%)	34 (12)	12 (12)	22 (12)	
Patient´s lack of compliance follow-up, n (%)	103 (36)	26 (25)	77 (43)	
GFR loss faster than expected, n (%)	54 (19)	31 (30)	23 (13)	
Patient´s related healthcare bureaucracy issues, n (%)	31 (11)	4 (4)	27 (15)	
Non-functional vascular access at start, n (%)	13 (10)	9 (9)	4 (2)	
Unknown	10 (3)	9 (8)	1 (0.4)	

Abbreviations: CKD, chronic kidney disease; NP, non-planned patients; ER+NP, early referral and non-planned patients; LR+NP, late referral and non-planned patients.

**Fig 2:**
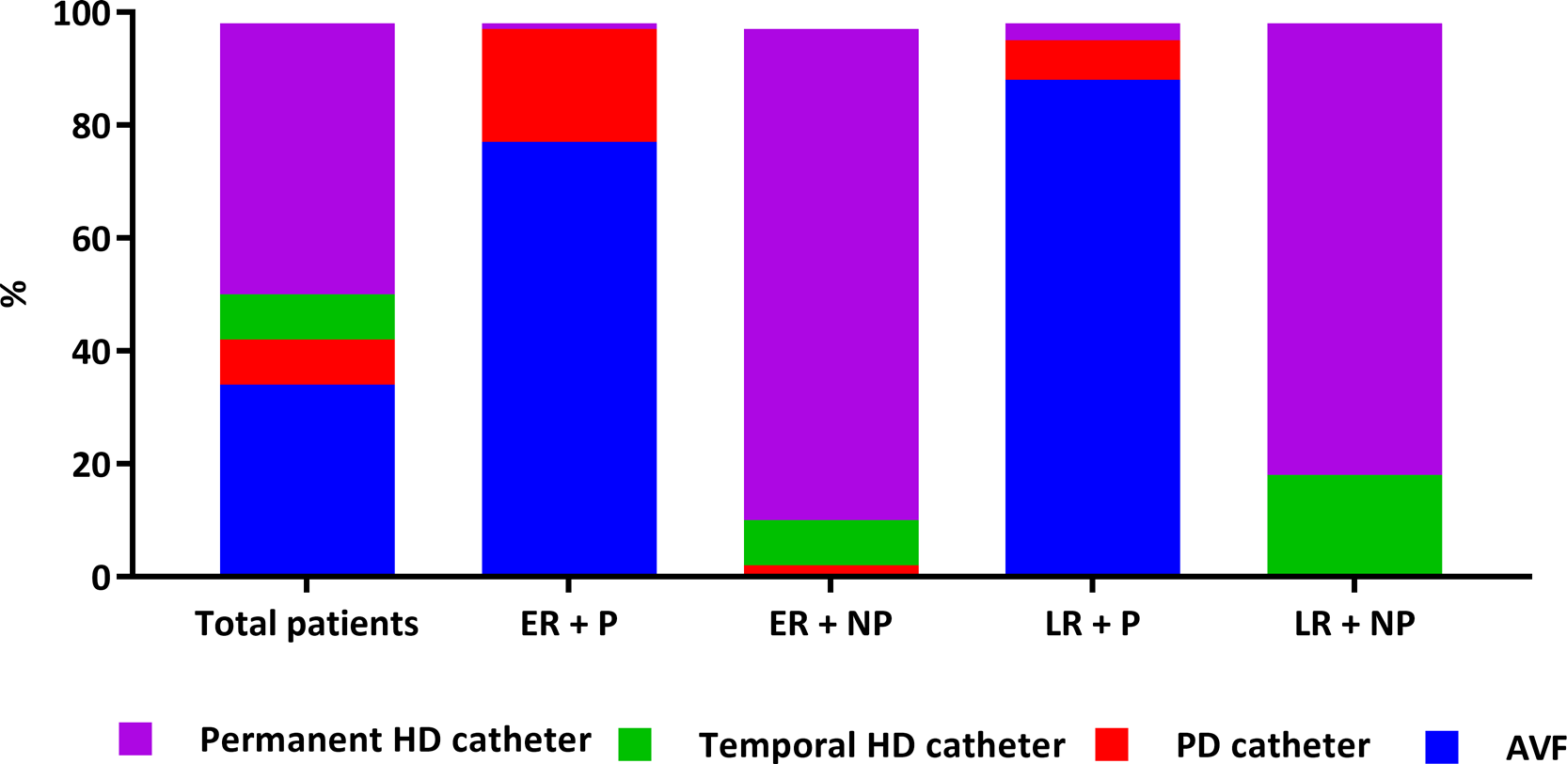
Type of dialysis access at first dialysis session accordingly with different studied subgroups. Abbreviations: ER+P, early referral and planned patients; ER+NP, early referral and non-planned patients; LR+P, late referral and planned patients; LR+NP, late referral and non-planned patients. PD, peritoneal dialysis; HD, hemodialysis; AVF, arterio-venous fistula. Figure represents a diagram of bars showing the different types of accesses at first dialysis session. Accesses were as follows for the total population: 34.5% AVF, 8% peritoneal catheter, 8.5% temporal hemodialysis catheter and 49% permanent HD catheter. For ER+P: 77% AVF, 21% peritoneal catheter, no temporal hemodialysis catheter and 2% permanent HD catheter. For ER+NP: 0.8% AVF, 2.6% peritoneal catheter, 9% temporal hemodialysis catheter and 88% permanent HD catheter. For LR+P: 89% AVF, 8% peritoneal catheter, no temporal hemodialysis catheter and 3% permanent HD catheter. For LR+NP: 0.4% AVF, 1% peritoneal catheter, 18% temporal hemodialysis catheter and 80% a permanent HD catheter.

316/547 (58%) started dialysis as NP and 113/316 (36%) of the NP patients were previously followed up, for at least 3 months, by nephrologists (54% of patients at an ICS clinic vs. 46% by referral nephrologists). Additionally, patients with NP start had worse clinical status at dialysis start and worse access management (Table 1 and Fig 2).

Factors associated with P start were evaluated by a multivariate logistic regression analysis and are described in Table 3. Factors were adjusted for age and gender.

**Table 3:** Multivariate logistic regression for planned versus non-planned dialysis start. Pseudo r2 = 0.26.

n = 547	Odds ratios and 95% CI	P
Age, years	1.00 (0.98–1.02)	0.97
Gender, female vs male	0.84 (0.52–1.33)	0.16
eGFR (MDRD 4), > 8.2 ml/min vs. ≤ 8.2 ml/min	2.72 (1.72–4.27)	0.001
Time from information to initiation of dialysis start, > 2 months vs. ≤ 2 months	4.84 (2.71–8.65)	0.001
Early referral vs late	2.12 (1.17–3.84)	0.03
Diagnosis, Other vs. vascular	0.34 (0.19–0.60)	0.001

More patients received education in the P (218/231, 94%) than in the NP group (218/316, 69%). At the time of modality information, P start patients had lower serum creatinine, longer predialysis follow-up and more patients were started on PD as RRT (p ≤0.01) (Table 4).

**Table 4:** Characteristics of patients with early referral (>3months) to Integrated Care Settings clinics follow-up according to planning of dialysis start.

Population	Total	P	NP	P-value
ER to ICS, n (%)	281 (100)	168 (60)	113 (40)	0.001
Median CKD follow-up before dialysis start (m.)	15.1 (3–65)	18.1 (5–65)	12 (0.9–83)	0.01
Median time of predialysis follow-up (m.)	6.7 (0.3–38)	8.2 (2–45)	4.9 (0–26.4)	< 0.001
Predialysis follow-up, n (%)	241 (86)	156 (93)	85 (75)	< 0.001
Serum creatinine at information (mg/dl)	4.9 (3–10)	4.5 (2.7–11)	6.0 (2.8–13)	< 0.001
Information on dialysis modalities, n (%)	241 (86)	160 (95)	81 (72)	< 0.001
Information provided consent signing, n (%)	144 (51)	88 (52)	56 (49.5)	< 0.001
Medical visits during predialysis follow-up, n		8 (2–27)	2 (0–14)	< 0.001
Hospitalizations during predialysis follow-up, n		2 (0–4)	1 (0–4)	< 0.001
PD as 1^st^ dialysis session, n (%)	37 (13)	34 (20)	3 (2.6)	< 0.001
PD as 1^st^ chronic RRT, n (%)	44 (16)	34 (20)	9 (8)	0.01

Values are median (10^th^ to 90^th^ percentile), or percentage. Abbreviations: P, planned dialysis start patients; NP, non-planned dialysis start patients; ICS, integrated care setting clinics; CKD, chronic kidney disease; (m.), months; RRT, renal replacement therapy; PD, peritoneal dialysis.

### Early Referrals

The group of ER + NP patients showed markedly lower indicators of quality care than ER+P patients as well as less use of PD (p<0.05) [Table 4]. On the other hand, in a multivariate logistic regression analysis, the ER+P group was associated with eGFR >8.2 ml/min (OR 2.64, p = 0.001) and with information provided >2 months before initiation of dialysis (OR 38.5, p = 0.001). The final model was adjusted for age, gender, renal etiology and eGFR.

### PD as RRT

**Fig 3:**
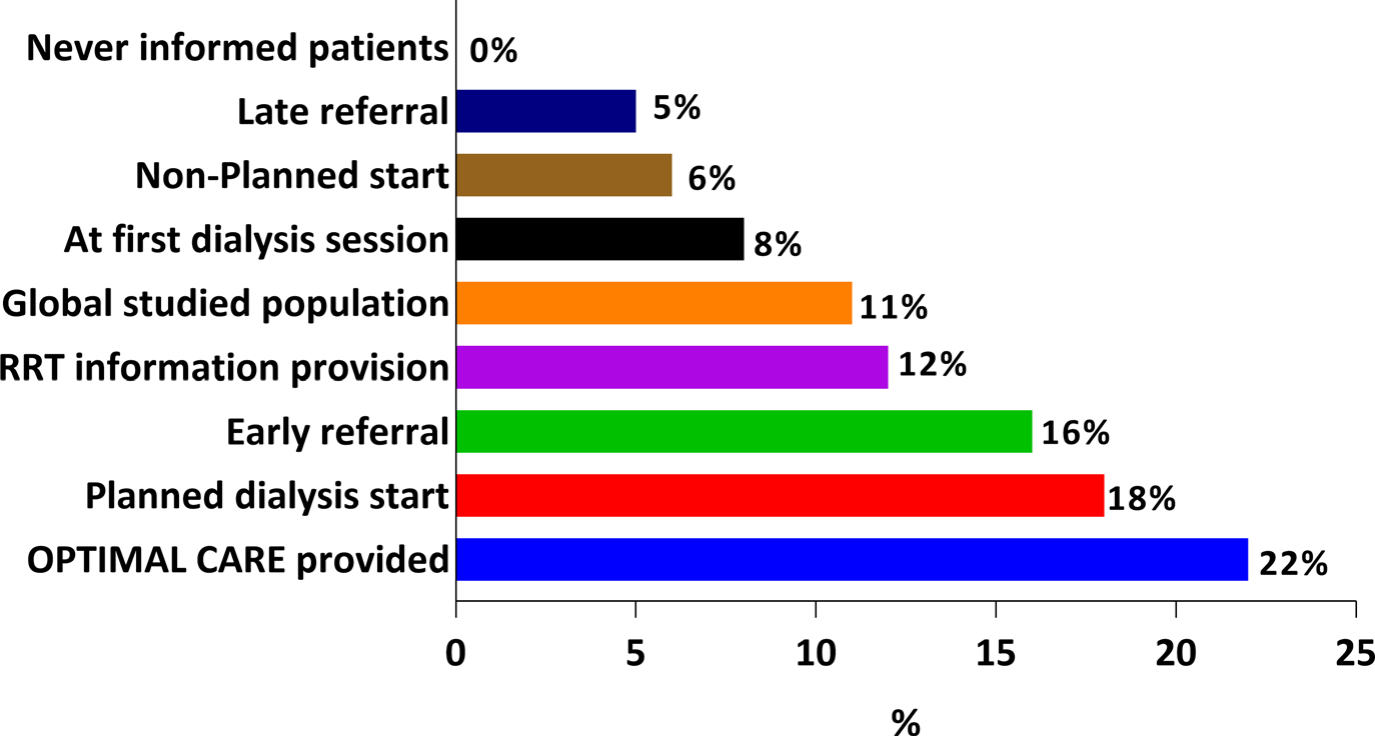
Peritoneal dialysis (PD) incidence (%) according with different studied subgroups. Maximum PD incidence was observed in the optimal care treated patients group being 22%. PD ranged 18% in the planned dialysis start, 16% in the early referred patients, 12% at modality information provision, 6% in the non-planned dialysis start, 5% in the late referral and no PD was observed if never previously informed. PD at the first dialysis session occurred in 8% and as first chronic RRT in 11% of the total studied population.

PD was performed as first dialysis modality in 8.2% of patients (n = 45), with 5/45 as unplanned start. On the other hand, 14 NP patients who started with HD and a central venous line were switched to PD in the next six weeks reaching a final PD incidence of 59/547 (10.7%) (Table 5 and Fig 3).

**Table 5:** Clinical characteristics according to initial RRT modality.

Population	All	HD	PD	P-value
n	547	488	59	
**Group: n (%)**				
ER+P	168 (31)	133 (27)	35 (59)	<0.001
ER+NP	113 (20)	104 (21)	9 (15)	
LR+P	63 (12)	58 (12)	5 (9)	
LR+NP	203 (37)	193 (40)	2 (17)	
Gender, Male, n (%)	332 (61)	298 (61)	34 (57)	0.67
**Age at dialysis start**				
< 35 years, n (%)	21 (4)	16 (4)	5 (8)	<0.001
35–50 years, n (%)	66 (12)	48 (10)	18 (30)	
51–65 years, n (%)	186 (34)	166 (34)	20 (34)	
66–75 years, n (%)	127 (23)	119 (24)	8 (14)	
> 75 years, n (%)	147 (27)	139 (28)	8 (14)	
**Cause of ESRD**				
Diabetes mellitus, n (%)	162 (30)	143 (29)	19 (32)	0.18
Glomerular, n (%)	64 (12)	52 (11)	12 (20)	
Inherited, n (%)	26 (4)	21 (4)	5 (8)	
Unknown, n (%)	52 (9)	49 (10)	3 (5)	
Others, n (%)	56 (10)	51 (10)	5 (8)	
Tubulo-interstitial, n (%)	62 (11)	57 (12)	5 (8)	
Vascular, n (%)	125 (23)	115 (24)	10 (17)	
Followed at initiation of CKD care by Nephrologist, n (%)	264 (48)	230 (47)	34 (58)	0.13
Time since initiation of CKD care to dialysis start (m.)	12.3	12.8	10.0	0.45
	(0.3–55)	(0.26–58)	(0.3–49)	
Patient followed in predialysis (GFR<30 ml/min), n (%)	332 (60)	288 (59)	44 (75)	0.02
Patient followed by specialized predialysis staff, n (%)	160 (29)	145 (30)	15 (25)	0.54
Patient educated in modalities, n (%)	436 (80)	382 (78)	54 (92)	0.02

Values are median (10^th^ to 90^th^ percentile), or percentage. Abbreviations: CKD, chronic kidney disease; ER+P, early referral and planned patients; ER+NP, early referral and non-planned patients; LR+P, late referral and planned patients; LR+NP, late referral and non-planned patients; HD, hemodialysis; PD, peritoneal dialysis; (m.), months; GFR, glomerular filtration rate.

PD incidence varied with age and patient subgroup (Fig 3). Patients who were not informed about RRT modalities never used PD. It is worthy to note that optimal care conditions had a big impact on the probability of PD as final RRT modality. Almost half of the PD patients (29/59, 49%) belonged to the optimal care patient group, whereas only 94/488 (19%) of HD patients did (p = 0.01).

### Type of dialysis access (vascular or peritoneal)

Access at first dialysis session is described in Fig 2. Serum creatinine and CCr 24h at the time of access request were better in the P than in the NP group [4.9 (3.1–10) mg/dl; 14 (7.9–15.8) ml/min vs. 5.7 (3.1–11.1) mg/dl; 9.7 (5–18.9) ml/min], (p<0.001).]

Patients starting (n = 316) with a temporal vascular catheter were progressively switched in the next six weeks to a different access: 49% into an AVF, 36% permanent vascular catheter, 5% with a peritoneal catheter and no grafts use.

## Discussion

In our multicenter, international experience most patients had medical follow ups since diagnoses of kidney disease. Almost half of the CKD care was provided by nephrologists. However, 49% of patients were referred late to our ICS clinics and 58% started dialysis in a NP manner, without a permanent dialysis access and/or in an emergency situation, most likely increasing morbidity, mortality and the cost of RRT [21,30–31]. To our knowledge, this is the first evaluation of type of referral, dialysis start and modality choice on RRT described in Eastern Europe.

Over the past several years, interest has evolved in evaluating the timing of nephrology referral in the predialytic stage of CKD as an important variable related to prognosis. Late referral to predialysis care and its quality may influence the selection of dialysis modality as well as the timing and planning of dialysis start [19,23,30–34]. The definition of the time factor “late” is somewhat arbitrary and varies in the literature, ranging from less than 1 month to less than 6 months follow-up before RRT is started.

Early referral to ICS was defined as at least a 3-month follow-up within the clinics’ care before starting RRT. However, at least one year is usually required to educate and optimize the preparation for RRT [13,32–34]. There are wide differences between different centers and countries in late referrals [35–36]. In Spain, Italy and France, data show that 20–25% of patients experienced late referrals, while higher figures are reported for other countries [36–42]. The relatively low involvement of nephrologists since initiation of CKD follow-up (48%) compared with other series [23] may partially explain the high level of late referral. Late referral may deprive the patient from treatment to prevent or delay CKD progression and access to kidney transplantation, and inevitably lowers the possibility of receiving education, as well as choice options [11,32,43].

Numerous factors may be involved in a NP start, although some are unpredictable and others unacceptable/undesirable: asymptomatic renal disease (unpredictable), inadequate diagnosis or treatment of CKD (unacceptable), unexpected rapid deterioration of renal function, socio-economic reasons, patients reluctant to initiate dialysis or whose physicians underestimate the potential benefits of dialysis, long waiting lists to attend a predialysis care unit (unacceptable), waiting list for performing vascular access (unacceptable or undesirable) and others [23,41–44]. To our knowledge only one earlier study has covered the real reasons behind an unplanned start especially in those patients previously followed by nephrologists [44]. In our study, patient-related reasons accounted for almost half of the causes behind a NP start.

It is striking that 21% of patients with NP start were previously followed-up for at least 3 months in our ICS clinics, but this period was considered insufficient to assure proper medical and emotional management and support, underlying a need to improve logistics at the time of referral. The high prevalence of late referred patients impacts the type of dialysis start and therefore the selection of dialysis modality. The large penetration of HD is higher for late referred patients and/or without previous follow-up. The fact that more patients who received information had a P start underlies the importance of patient empowerment for better control of risk factors, fluid overload and treatment compliance [33].

Our data indicate that there is an opportunity for improvement, as only 23% of patients had optimal care considered as followed-up at ICS clinics by nephrologists for >1 year, educated on dialysis modalities and with a planned dialysis start. Similarly to other series, choice of PD is more frequent with optimal care, confirming that PD patients are generally better informed, more conscious of their disease, know more about other RRT modalities and are more prone to recommend their therapy to other patients or even be more actively laboring [45–46].

It is also remarkable that modality information and renal education were widely provided regardless of late referral and NP dialysis start, and that a large group of patients signed consents as information was provided and at dialysis start in accordance with recent international regulations [47–48]. Nevertheless, the completeness and balance of information may have been overestimated, as clinics were free in the way they were delivering information and/or education to patients. This may be considered a limitation of the study. Proper information provision should have covered a structured process based in decision-making aids guidelines [49–50]. A well-balanced presentation of all therapeutic options is usually associated with a higher selection of PD as first therapy [19,21,23,34,50] and, indeed, up to 50% of patients without medical contraindications for PD or HD selected PD [34,51–54]. Patients with no previous PD information could not choose PD. Thus, we may expect higher rates of PD in the near future after increasing the number of specialized predialysis care staff at our clinics and streamlining the choice process [12,21,34,50–51]. In this regard, we are planning to assess the impact of implementing the routine use of “decision-making aids” from 2014 [55]_._

Most authors have described better outcomes, longer survival, higher proportion of planned dialysis start and more PD choice for patients switched into specialized predialysis programs than if followed-up by a general nephrologist [23,50,56]. In this regard, we did not observe significant differences in terms of PD take on, probably related with the low number of clinics staffed by a specialized predialysis nephrologist and nurse during 2012. The shortage of nephrologists that some of these countries face does not permit a universal predialysis care specialization.

## Conclusions

Although patients were frequently followed-up from the time of CKD diagnosis, referral patterns to ICS clinics have not been fully successful in Eastern Europe. Unplanned start was frequent and may explain the low frequency of PD. Despite the high rate of late referral, information and education were widely provided but probably not consistently structured and not long enough in duration due to the late referral. Measures such as implementation of referral patterns, reinforcement of predialysis staff specialization and routine use of decision-making aids may facilitate optimal care, improving well-being and planning of RRT start as well as increased PD use.
